# Fatal Septic Shock in a Patient with Hemophagocytic Lymphohistiocytosis Associated with an Infectious Mononucleosis

**DOI:** 10.1155/2018/9756050

**Published:** 2018-09-25

**Authors:** Giorgio Berlot, Ariella Tomasini, Lorenzo Zandonà, Eugenio Leonardo, Rossana Bussani, Nadia Zarrillo

**Affiliations:** ^1^University of Trieste, Department of Anesthesia and Intensive Care, Cattinara Hospital, 447, 34149, Trieste, Italy; ^2^University of Trieste, Department of Pathology, Cattinara Hospital, 447, 34149, Trieste, Italy; ^3^Caserta Hospital, Department of Anesthesia and Intensive Care, Italy

## Abstract

The authors describe the case of a young woman who developed a clinical pictures resembling a septic shock-related multiple organ dysfunction syndrome a couple of months after having been diagnosed suffering from a hemophagocytic lymphohistiocytosis associated with an infectious mononucleosis. Despite the aggressive treatment, which included antibiotics, vasopressors, IV immunoglobulins, and the use of an extracorporeal device aimed to remove mediators released both during sepsis and the cytokine storm determined by the hemophagocytic lymphohistiocytosis, the patient died. At the autopsy, an extremely uncommon aggressive lymphoma of Epstein-Barr virus-positive T-lymphocytes with systemic involvement was discovered.

## 1. Introduction.

Although most patients with infectious mononucleosis (IM) recover without major clinical sequelae, a number of harmful complications can occur, including hepatitis, splenic rupture, and hematological abnormalities [1]; among the latter, the hemophagocytic lymphohistiocytosis (HLH) is particularly harmful because it is characterized by the massive production and release of inflammatory mediators caused by the uncontrolled activation of T-lymphocytes, NK-cells, and macrophages; the ensuing clinical picture resembles septic shock and can determine a multiple organ dysfunction syndrome (MODS) [2–4]. The HLH is increasingly recognized in patients admitted to the Intensive Care Units (ICU) due to different conditions including hematologic and solid tumors, autoimmune diseases, and bacterial and viral infections. [4–6]; as far IM-associated HLH is concerned, its occurrence has been ascribed to the persistence of the Epstein-Barr virus (EBV) in the tissues and the outcome is particularly poor [3].

Independently of the underlying triggering cause(s) the differential diagnosis of secondary HLH is difficult and relies mainly on a high index of suspicion and a number of hematological and biochemical criteria basically derived from those used in the pediatric form which is mostly caused by genetic abnormalities [7], making their use less useful in the acquired form (Table 1).

Here we describe the case of a patient who developed a rapidly evolving MODS a couple of months after the occurrence of an IM complicated by HLH.

## 2. Case Description

A 24-year-old woman was admitted to the Department of Infectious Disease with fever (T = 38,3°C) which was attributed to a relapse of a IM-HLH occurring about two months prior to the current hospitalization. At that time, the diagnosis of IM was confirmed by the presence of EBV DNA in the bloodstream and the diagnosis of HLH was suspected on the basis of the clinical findings such as persisting fever, the enlargement of the liver and the spleen as well as of blood abnormalities including pancytopenia, abnormally elevated values of ferritin (> 15.000 mcg/ml), triglycerides (789 mg/dl), and liver enzymes (AST 175 U/L, AST 120 U/L); a bone marrow biopsy confirmed the presence of hemophagocytosis and viral RNA in many cells by means of in situ hybridization (EBER) [7]; a genetic screening excluded the presence of gene mutations associated with HLH. During that hospitalization, she received steroids, etoposide, rituximab, cyclosporine, granulocyte-stimulating factor, and intravenous immunoglobulins (IvIg) and was ultimately discharged 38 days after the initial admission without viral DNA detectable in the bloodstream. During her stay at home, which lasted 3 weeks, the patient received prednisone, cyclosporine, trimethoprim-sulphametoxazole and acyclovir through a peripherally inserted central venous catheter (PICC). Thirty-six hours after the current admission during which rituximab was added to the ongoing treatment she was transferred to the ICU due to the deterioration of the consciousness, arterial hypotension, and fever. At the ICU admission, the patient presented high fever (40,5°C), disseminated intravascular coagulation, arterial hypotension, and acute kidney injury requiring renal replacement therapy (RRT): a methicillin-resistant* Staph. aureus* (MRSA) was isolated from the blood cultures and a septic shock-related MODS possibly in association with a cytokine storm caused by the HLH were hypothesized; the patient was intubated and mechanically ventilated and treated with IV vasopressors at incremental doses, IV vancomycin, meropenem, and caspofungin; the PICC was removed and replaced with a central venous catheter; as the patient remained unresponsive to the treatment, a Coupled Plasma Filtration and Adsorption treatment (CPFA, LYNDA®, Bellco, Mirandola, Italy) that aimed at removing the inflammatory mediators was added to the RTT along with the IV administration of IgM and IgA-enriched IvIg (Pentaglobin®, Biotest; Dreieich, Germany). Despite this increasingly aggressive approach, the MODS further worsened and the patient died 18 hours after the ICU admission.

**Figure 1:**
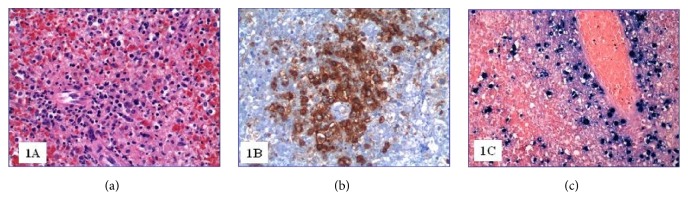
(a) Spleen tissue (H&E 200x); (b) CD8 positive spleen lymphocytes (200x); (c) spleen EBV positive cells (EBER, 200x).

**Figure 2:**
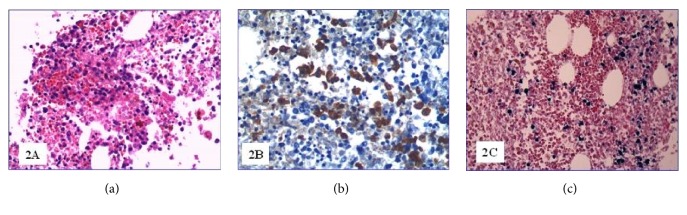
(a) Bone marrow pleomorphic lymphocytes (H&E 200x); (b) CD8 positive bone marrow lymphocytes (200x); (c) bone marrow EBV positive lymphocytes (EBER, 200x).

**Figure 3:**
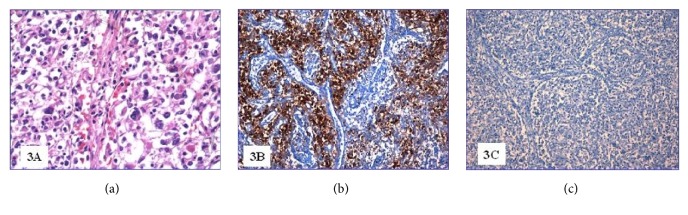
(a) Mediastinal lymph node (H&E 200x); (b) CD8 positive lymph node lymphocytes (200x); (c) PAX-5 negative lymph node lymphocytes (200x).

At the autopsy, the liver and the spleen appeared enlarged, weighting 3110 g and 1230 g, respectively. Microscopically, the spleen showed lymphocyte depletion and the scattered necrosis of Malpighi's follicles (Figure 1(a)) combined with subversion of the general architecture due to a proliferation of T-lymphocytes (CD3+) with predominant expression of CD8 (Figure 1(b)). The lymphocyte population B (CD20+, PAX-5+) was virtually absent, while there was a significant increase in monocyte-macrophage component sometimes associated with hemophagocytosis. The EBER highlighted several lymphocytes with integrated EBV RNA in the nucleus (Figure 1(c)). The liver showed several perivascular infiltrates of polymorphic lymphocytes likewise those found in the spleen and many monocyte-macrophage cells (CD14+, CD64+). In the bone marrow there were multiple lymphocyte with polymorphic or abnormal nucleuses (Figure 2(a)) that appeared to be almost exclusively CD8+ T-lymphocytes (Figure 2(b)). Most of these cells were EBER positive (Figure 2(c)); B-lymphocytes (CD20+, PAX-5+) were almost absent while there was an expansion of the monocyte-macrophage series (CD14 +, CD64 +). The three hematopoietic lines appeared contracted but with preserved maturation. Mediastinal lymph nodes showed a diffuse proliferation of medium/large sized lymphoid elements, sometimes with single or multiple polymorphic nuclei (Figure 3(a)). The lymphocyte population was composed by CD3+ CD8+ T-lymphocytes with a limited presence of lymphocytes CD3+ CD4+ and absence of B cells (CD20 +, PAX-5 +).

## 3. Discussion

Different pathologic conditions, including macrophage activation syndrome (MAS), adult onset Still's disease (AOSD), catastrophic antiphospholipid syndrome (cAPS), and septic shock share a similar clinical presentation characterized by fever, arterial hypotension, and multisystem involvement due to the action of a number of inflammatory mediators [8]. This overlap of symptoms makes the diagnosis elusive and the appropriate approach can be delayed in the absence of other information. This difficulty is substantial because the treatments differ widely among the various clinical entities: as an example, in the AOSD, MAS, and CAPS, the administration of immunosuppressant agents is warranted [6, 9] but is strongly contraindicated in septic shock whose treatment is based on the prompt administration of large-spectrum antibiotics [10], which in many ICUs is combined with the administration of eIg and the use of blood purification techniques. This uncertainty can be particularly represented in hematological patients, in whom the onset of MODS can represent the final common pathway of different conditions, including immunosuppression-related sepsis, the cytokine release syndrome induced by novel cancer immunotherapies, and HLH [8, 11, 12]. Moreover, the proposed diagnostic criteria could not help in a critically ill patient, as none of them is sensible and sensitive enough and/or they are time-consuming: as an example, a recent study demonstrated the even extremely elevated blood ferritin levels were not predictive of LHL in adult patients [6]. Indeed, the described patient lied in the grey area in which an inappropriate approach means the difference between life and death. To overcome the risks associated with a wrong diagnosis and treatment we hypothesized that a septic shock was superimposed to a relapsed HLH and added to the already running therapies the CPFA aiming to remove the inflammatory mediators involved in both circumstances. Unfortunately, also this approach failed to modify the fulminant clinical course and the patient ultimately died. Moreover, it has been hypothesized an increased virulence and/or a reduced sensitivity to vancomycin in MRSA causing infections in patients with solid or hematologic malignancies [13].

The autoptical findings demonstrated that the septic shock occurred concomitantly with an aggressive lymphoma with multiorgan involvement; actually, it is likely that long-term treatment with immunosuppressant agents, albeit indicated for the treatment of the HLH, may have determined the evolution of the underlying chronic active EBV infection towards the aggressive T-cell lymphoma with systemic involvement. This is an extremely uncommon finding, as the tropism of EBV for B-lymphocyte has been associated with B-cells cancers, including Burkitt, Hodgkin, and diffuse large cell lymphomas [14, 15]; conversely EBV-positive T-cell lymphoma is exceedingly uncommon in western populations and has been attributed either to a possible antiapoptotic action exerted by the viral DNA hosted in the infected T-cells and/or to signals transmitted by some viral proteins such as Latent Membrane Protein 1 causing the continuous stimulation of members of the TNF receptors [2, 16]. Actually, it appears that in patients with EBV-related infections the chronic persistence of an elevated viral load represents a risk factor for HLH as well for other severe hematologic complications, including disseminated intravascular coagulation and lymphoma [2, 14]. In the described patient, however, viral DNA was absent at the discharge from the previous admission and this finding contrasts with the massive presence of EBV in all tissues demonstrated at the autopsy. It is likely that the immunosuppressant agents given to treat the HLH and to prevent its relapse acted as a double-edged sword: from one side they abated the hemophagocytosis, which was present only marginally at the post mortem examination of the bone marrow but from the other one triggered the reactivation of the EBV and the rapidly developing lymphoma.

## 4. Conclusions

The occurrence of EBV infection-associated HLH and the prevention of its relapse consists in the prolonged administration of immunosuppressant agents possibly associated with bone-marrow transplantation. Similar to other conditions associated with the decrease of the immune capabilities, this treatment exposes the patients to infectious complications, including viral reactivation and overwhelming bacterial infections. The occurrence of aggressive hematologic cancers is uncommon but must be kept into consideration in patients with HLH admitted with conditions resembling septic shock.

## Figures and Tables

**Table 1:** Diagnostic criteria of HLH.

Molecular diagnosis consistent with HLH.	

Or 5 of the following criteria	
(1) Fever	
(2) Splenomegaly	
(3) Cytopenia affecting ≥ 2 lineages	
(a) Hemoglobin < 9g / dl	
(b) Platelets < 90.000 / ml	
(c) Neutrophils < 1000 /ml	
(4) Hypertrigliceridemia and/or hypofibrinogemia	
(a) Triglycerides > 265 mg/dl	
(b) Fibrinogen < 150 mg/dl	
(5) Hemophagocytosis in bone marrow, spleen or nodes	
(6) Low/absent NK cell activity	
(7) Ferritin ≥ 500 mcg/ml	
(8) sCD25 (sIL2R) ≥ 2400U/ml	

## References

[1] Luzuriaga K., Sullivan J. L. (2010). Infectious mononucleosis. *The New England Journal of Medicine*.

[2] Tawfik K., Liron Y., Ayman A. R., Schneider R., Wolf D., Ronen L. (2015). A heart breaking case of rapidly developing severe hemophagocytic syndrome secondary to chronic active EBV infection; a case report and review of the literature. *Journal of Clinical Virology*.

[3] Buyse S., Teixeira L., Galicier L. (2010). Critical care management of patients with hemophagocytic lymphohistiocytosis. *Intensive Care Medicine*.

[4] Janka G. E., Lehmberg K. (2014). Hemophagocytic syndromes—an update. *Blood Reviews*.

[5] Gauvin F., Toledano B., Champagne J., Lacroix J. (2000). Reactive hemophagocytic syndrome presenting as a component of multiple organ dysfunction syndrome. *Critical Care Medicine*.

[6] Schram A. M., Berliner N. (2015). How I treat hemophagocytic lymphohistiocytosis in the adult patient. *Blood*.

[7] Neparidze N., Lacy J. (2014). Malignancies associated with Epstein-Barr virus: Pathobiology, clinical features and evolving treatments. *Clinical Advances in Hematology & Oncology*.

[8] Rosário C., Zandman-Goddard G., Meyron-Holtz E. G., D'Cruz D. P., Shoenfeld Y. (2013). The hyperferritinemic syndrome: macrophage activation syndrome, Still's disease, septic shock and catastrophic antiphospholipid syndrome. *BMC Medicine*.

[9] Jordan M. B. (2018). Emergence of targeted therapy for hemophagocytic lymphohistiocytosis. *The Hematologist*.

[10] Rhodes A., Evans L. E., Alhazzani W. (2017). Surviving sepsis campaign: international guidelines for management of sepsis and septic shock: 2016. *Intensive Care Medicine*.

[11] Shimabukuro-Vornhagen A., Gödel P., Subklewe M. (2018). Cytokine release syndrome. *Journal for ImmunoTherapy of Cancer*.

[12] Azoulay E., Pène F., Darmon M. (2015). Managing critically Ill hematology patients: time to think differently. *Blood Reviews*.

[13] Blennow O., Ljungman P. (2016). The challenge of antibiotic resistance in haematology patients. *British Journal of Haematology*.

[14] Rezk S. A., Weiss L. M. (2007). Epstein-Barr virus-associated lymphoproliferative disorders. *Human Pathology*.

[15] Lehmberg K., Ehl S. (2013). Diagnostic evaluation of patients with suspected haemophagocytic lymphohistiocytosis. *British Journal of Haematology*.

[16] Park S., Ko Y. H. (2014). Epstein-Barr virus-associated T/natural killer-cell lymphoproliferative disorders. *The Journal of Dermatology*.

